# Supplementary material for: Specific inhibition and disinhibition in the higher-order structure of a cortical connectome
Source: Cereb Cortex. 2024 Nov 11;34(11):bhae433. doi: 10.1093/cercor/bhae433 (PMC11551764; doi:10.1093/cercor/bhae433)
Supplement: Inhibition_in_MICrONS_supp_bhae433 [file inhibition_in_microns_supp_bhae433.pdf]

SUPPLEMENTARY INFORMATION

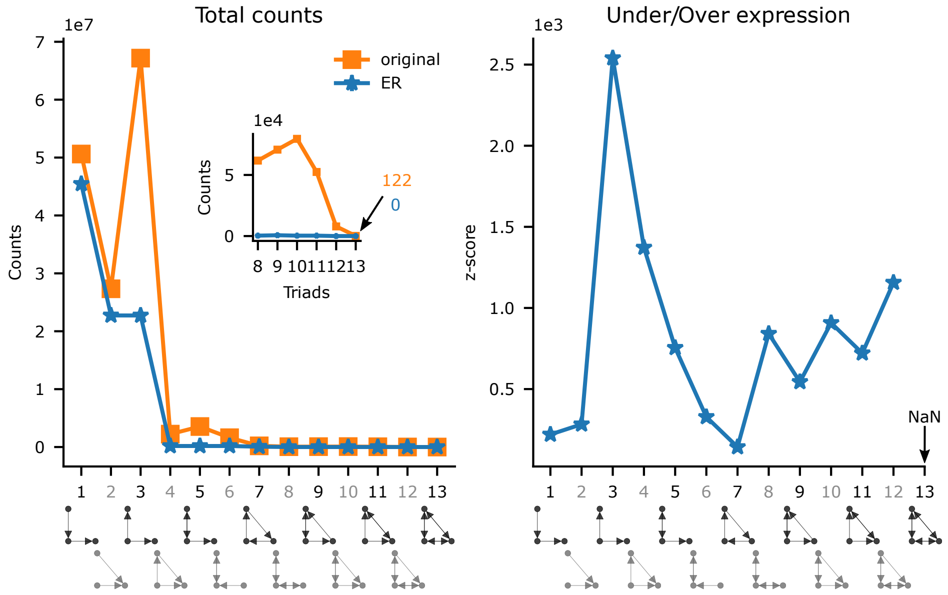

Supplementary Figure 1. Left, triad motif counts. In blue, counts for the central subnetwork of the MICrONS dataset. In orange mean and std of the counts of 30 Erdős–Rényi controls. Right, MICrONS counts z-scored with respect to the distribution of the counts on the Erdős–Rényi controls.

		Class					
		**23P**	**4P**	**5P_IT**	**5P_PT**	**6CT**	**6IT**
Position	**0**	0.91%	31.5%	25.7%	33.8%	7.7%	0.3%
	**1**	9.2%	30.6%	26.1%	30.9%	0.2%	3.0%
	**2**	12.2%	33.6%	18.5%	30.6%	0.0%	5.0%
	**3**	11.9%	30.2%	19.6%	30.3%	0.1%	7.9%
	**4**	8.9%	29.6%	8.5%	35.2%	0.1%	17.7%
	**5**	8.8%	23.2%	8.5%	44.3%	0.0%	15.2%
	**6**	7.2%	23.0%	7.1%	39.1%	0.0%	23.0%
Neurons		28.7%	29.3%	12.3%	3.5%	16.6%	9.5%
Out-edges		27.1%	33.2%	16.2%	5.7%	10.3%	7.5%
In-edges		34.4%	31.1%	13.4%	6.1%	9.0%	6.0%

Supplementary Table 1. 6-dimensional simplex composition in terms of cell types. Below divider: For comparison, the fractions of neurons of each cell type, and the fractions of outgoing and incoming edges associated with each cell type.

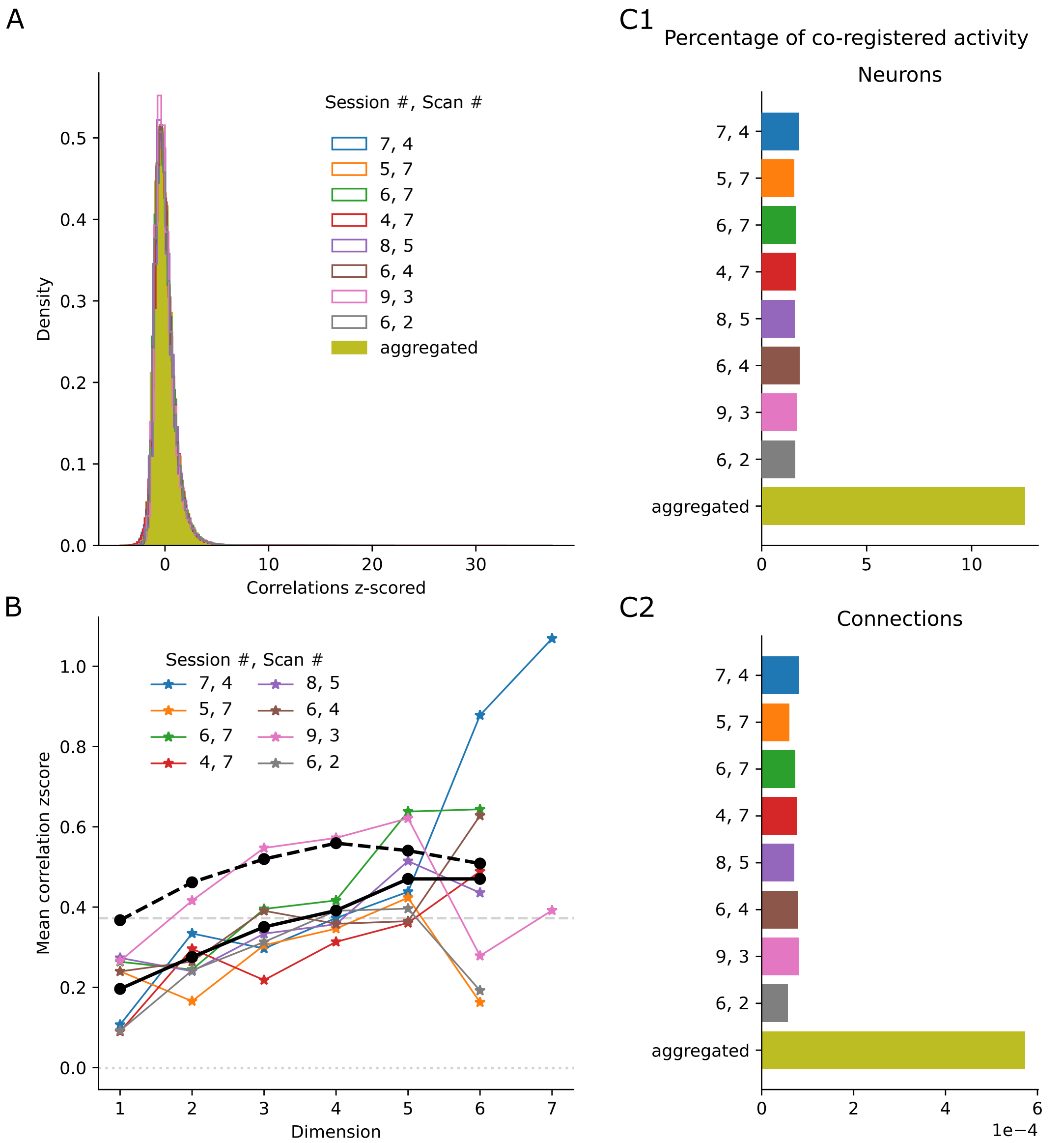

Supplementary Figure 2. Activity per repetition in the MICrONS data set. A: Histograms of z-scored correlations for all repetitions. B1: Percentage of neurons with co-registered activity within the whole circuit in each repetition or aggregated across repetitions. B2: As B1 but for connections. C:Correlations of the activity (z-scored) of pairs of neurons against their simplex membership. The x-axis indicates the maximum dimension over simplices the connection participates in. Black lines: mean values over recording sessions. Black dashed line: values when only the last pair in a simplex is considered. Grey dashed line: Overall mean for connected pairs.

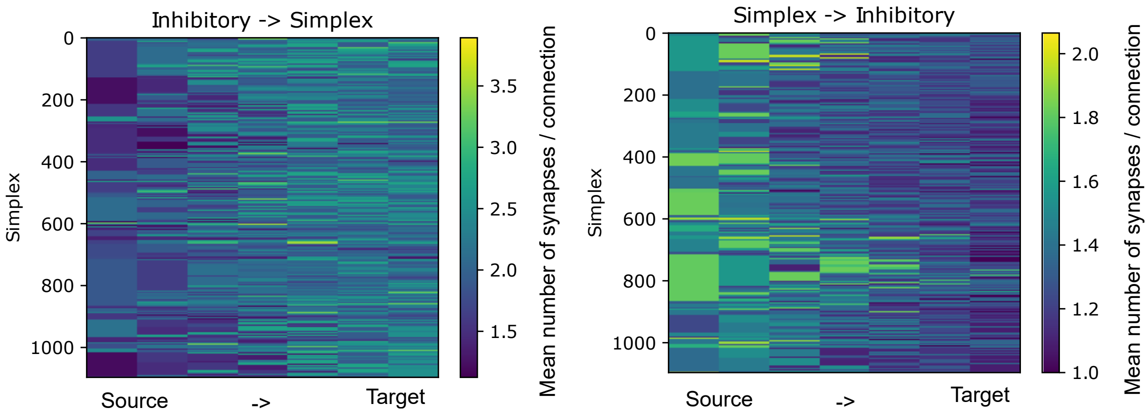

Supplementary Figure 3. Inhibitory connectivity from / to 6-d simplices as in Fig. 3C, but instead of the degree (number of connections) we show the number of synapses per connection.

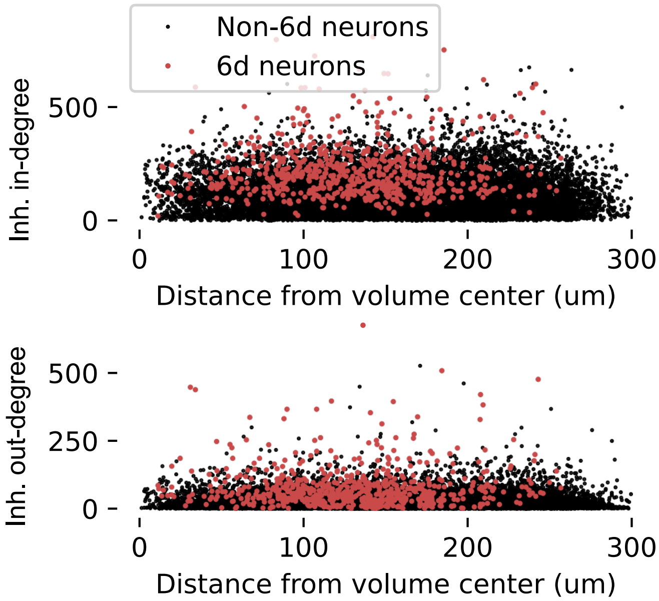

Supplementary Figure 4. Distance from the volume center against inhibitory in- and out-degree for neurons in the central subnetwork of MICrONS. For members of the 6-core (red) and non-members (black).

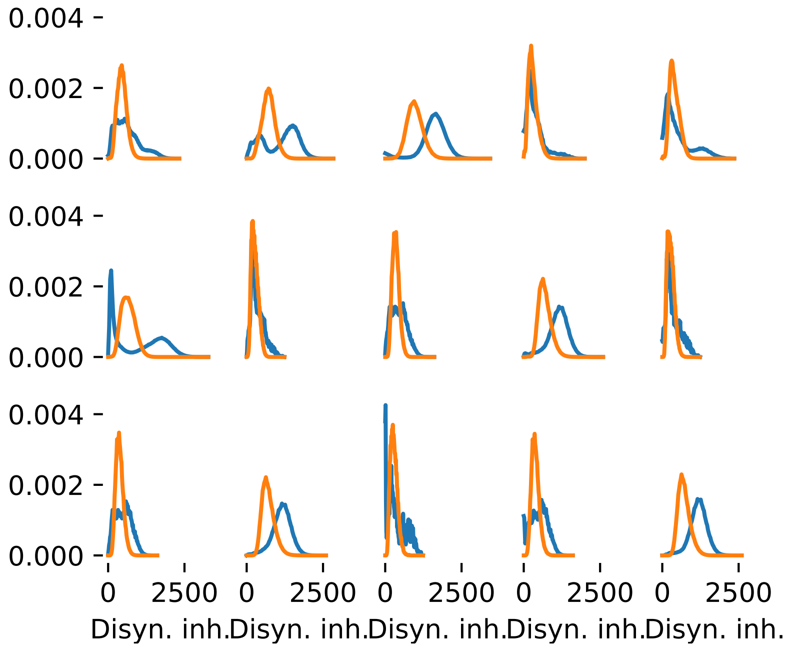

Supplementary Figure 5. Disynaptic inhibition strength comparing the data (blue) against a control (orange) as in Fig. 4C for all subnetworks of MICrONS.

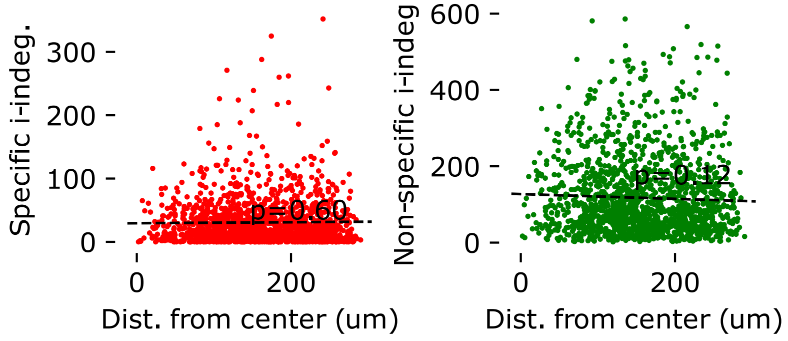

Supplementary Figure 6. Distance from the volume center against inhibitory in-degree of inhibitory neurons in the central subnetwork of MICrONS. Left: from inhibitory neurons with a significant targeting preference for other inhibitory neurons. Correlation is non-significant (pearsonr: 0.013; p=0.60) Right: from neurons without or with weak targeting preference. Correlation also non-significant (pearsonr: -0.04; p=0.12).

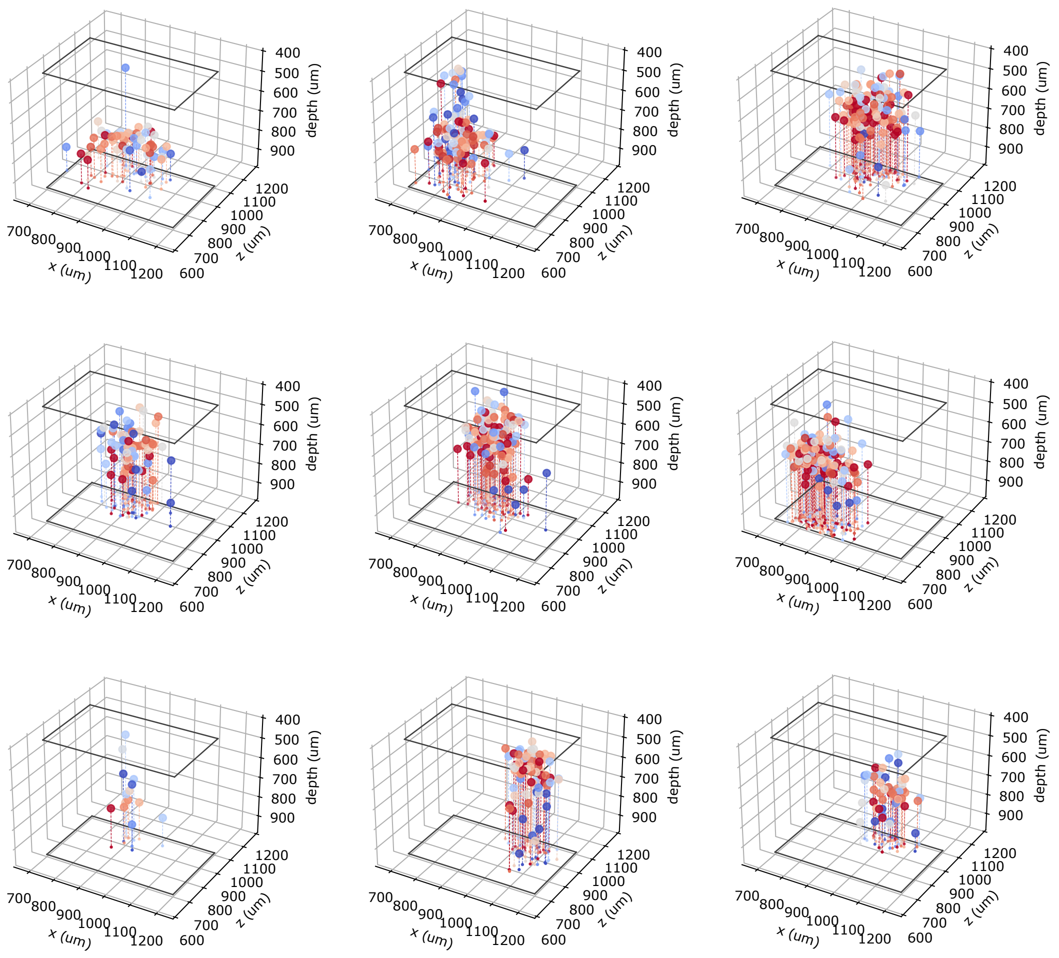

Supplementary Figure 7. Spatial locations of neurons in source groups as in Fig. 6B. Groups 0-8 of Fig. 5B ordered from left to right, and top to bottom.

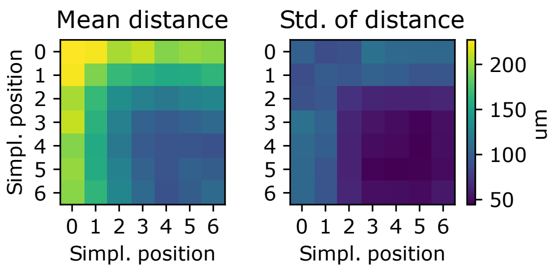

Supplementary Figure 8. Pairwise distances of neurons in the same source group against their simplex positions. Given a pair of simplices in the same source group, we can calculate the distances between the neuron in position i of one simplex and the neuron in position j of the other, for all combinations of i and j. The plot shows the mean (left) and standard deviation (right) over all pairs of simplices in the same source group, for the source groups in Fig. S 7. Instances where neurons i and j were the same neuron, due to simplex overlap, were ignored.

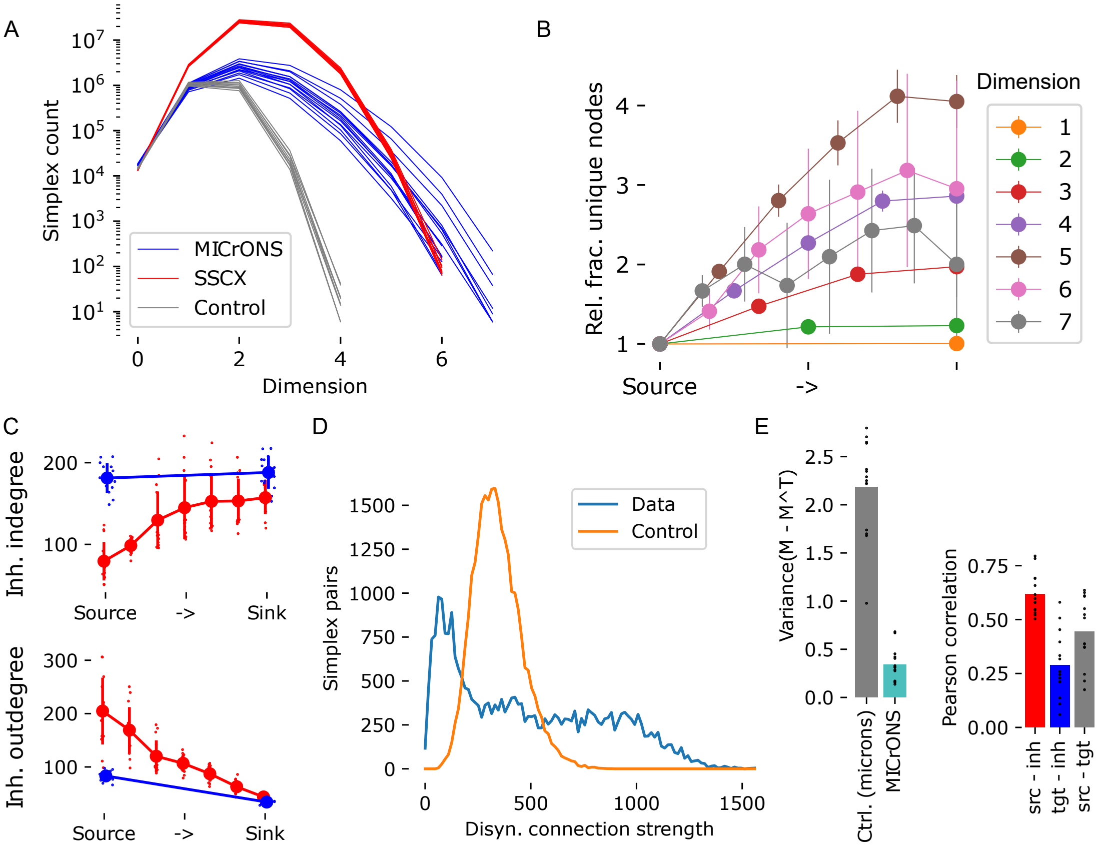

Supplementary Figure 9. Repeating analyses for a later, more proofread version of the MICrONS dataset. A: As Fig. 1D. B: As Fig. 2C1. C: As Fig. 3D, top. D: As Fig. 4C, top. E, left: As Fig. 4D, left. E, right: As Fig. 4H, left.

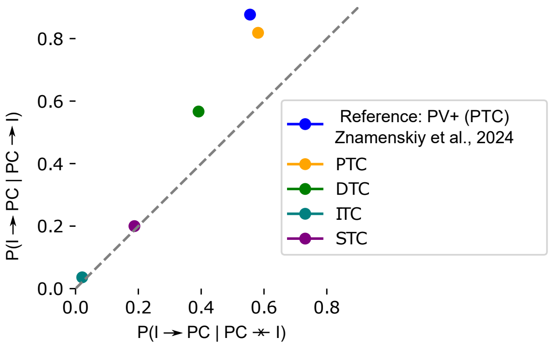

Supplementary Figure 10. Comparing analyzed connectivity in MICrONS to recent experimental results. Znamenskiy et al. (2024) found an overexpression of reciprocal connectivity between PV-positive neurons and pyramidal cells in layer 2/3 of mouse visual cortex. We characterize the overexpression by contrasting the inhibitory to PC connection probability where the PC is not connected to the inhibitory neuron (x-axis) with the connection probability where the PC is connected (y-axis). This is conducted for the four classes of inhibitory neurons characterized by Schneider-Mizell et al. (2023); the “PTC” class corresponds tentatively to PV-positive neurons. The grey line indicates identity, i.e. no bias for or against reciprocal connectivity.

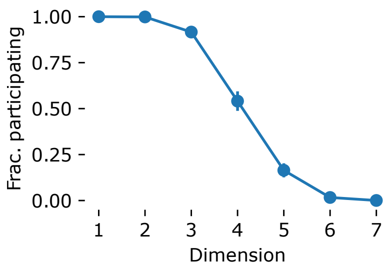

Supplementary Figure 11. Fractions of excitatory neuron that participate in at least one simplex of the indicated dimensions.
